# The impact of family and friend support of physical activity on the participation in physical activity within Indigenous individuals in Saskatoon

**DOI:** 10.3389/fspor.2024.1476949

**Published:** 2024-11-13

**Authors:** Nisha K. Mainra, Avery K. Ironside, Shara R. Johnson, Tayha T. Rolfes, Muqtasida A. Fatima, Kendra Melanson, Heather J. A. Foulds

**Affiliations:** College of Kinesiology, University of Saskatchewan, Saskatoon, SK, Canada

**Keywords:** First Nations, Métis, social support, exercise, physical activity

## Abstract

**Introduction:**

Social support within Indigenous worldviews is recognized as a component of health and has been associated with physical activity and sedentary behaviour. Physical Activity (PA) is a modifiable behaviour that can help reduce risks of disease and benefit many dimensions of health. The purpose of this study was to compare the physical activity of Indigenous adults in Saskatchewan with and without family/friend support of PA.

**Methods:**

The Family Influence on Physical Activity questionnaire was used to record the extent and forms of family/friend support of PA. The Godin Shepard Leisure Exercise Questionnaire (GSLEQ) was used to measure weekly PA, including moderate PA (MPA), vigorous PA (VPA), exercise frequency (WEF) and GSLEQ scores.

**Results:**

Indigenous participants overall, First Nations and Cree participants specifically with family/friend involvement in PA reported greater weekly WEF and GSLEQ scores. Indigenous participants overall and First Nations participants specifically with family and friends who watch them engage in PA reported greater weekly VPA and GSLEQ scores. Among First Nations and Cree participants specifically, those with family/friends encouragement of PA reported greater weekly VPA, WEF and GSLEQ scores. Furthermore, First Nations participants with active family/friends reported significantly greater weekly VPA (36.8 ± 51.5 min·week^−1^ vs. 80.2 ± 108.5 min·week^−1^; *p* = 0.01). Greater weekly VPA was found in Métis participants with family/friend involvement in PA and with family/friends who watch them engage in PA (67.6 ± 59.6 min·week^−1^ vs. 29.5 ± 40.8 min·week^−1^; *p* = 0.01).

**Discussion:**

Social support, specifically family/friends involvement, has a significant impact on Indigenous participation in PA.

## Introduction

Canada is inhabited by various distinct and diverse populations, including Indigenous Peoples who are the original inhabitants of these lands, and comprise 5% of the population of Canada (1). This encompasses First Nations, Inuit, and Métis Peoples of Canada (1). Each distinct Indigenous nation in Canada has their own homeland or traditional territory within the lands of Canada, though the majority of Indigenous Peoples do not reside within their reserve lands (2). Diversity of Indigenous communities and nations is significant, while urban centres and communities such as university settings serve Indigenous populations including diverse identities, histories, and cultures, with identities primarily drawn from Indigenous communities in the province of the university (1, 3).

Indigenous Peoples historically were a healthy population, with their health and wellness reflecting wholistic balance of the physical, mental, emotional, and social dimensions (4, 5). This balance also included strong family relations, community support and cultural connections (5). Cultural connectedness, a measure of attachment, sense of belonging, and group identification is a social factor that uniquely affects Indigenous Peoples’ health (6, 7). Current and historical acts of colonization and assimilation, including land displacement, residential schools, culture suppression, and the resulting intergenerational trauma experienced by Indigenous Peoples, have led to greater health disparities compared to non-Indigenous Peoples (8, 9). These health inequities include disproportionally higher prevalence of chronic diseases such as obesity, hypertension and cardiovascular disease (8, 10). While the general population in Canada have been able to reduce their risk of chronic diseases through behaviour and lifestyle changes including diet, tobacco use and physical activity (PA), the same is not true for Indigenous Peoples (8, 11).

Physical activity is one of the most modifiable behaviours to combat chronic diseases and is a significant contributor to health and wellbeing (12, 13). Additional benefits of PA include improving social and emotional wellbeing, which can lead to reductions in depression, stress, and anxiety, reduce social isolation, and increase feelings of wellbeing (14). Within the past 50 years, the health disparities between Indigenous and non-Indigenous populations have increased in part due to growing levels of inactivity and sedentary behaviour, which is influenced by a variety of factors, such as a lack of infrastructure close to reserve lands (15). In the general population, PA is influenced by many factors, including income, education, personal health, social support, community, and family influences (16). There is evidence currently indicating that factors such as cultural connections and social support play important roles in Indigenous Peoples’ PA and sedentary behaviour (17–19). There is, however, little understanding of the role of family/friend support for PA on Indigenous Peoples’ PA.

It is important to understand the role family support plays in participation in PA within Indigenous communities. Family from an Indigenous perspective is much broader than most Western definitions and includes immediate, extended, and unrelated community members who play significant roles in the lives of individuals (20). In some Indigenous cultures there is no word for family, and this is represented by “the community” (20). The Indigenous concepts of family are therefore influenced by social relations, support systems, language, childrearing practices and location (20). Families are often the primary source of social capital and the main providers of mental health care (20, 21). Research indicating that this extensive family support is essential to Indigenous welfare (20, 21). Family support is not only a crucial aspect of Indigenous ways of life but is also critical to community and individuals’ involvement and participation in various health promoting activities (22). Physical activity mentors, individuals who guide, teach, and support individuals in physical activity behaviour in a personal and peer-support type of relationship have been successful in supporting improved physical activity for older adults, and mentors in education and nutritional capacities have supported improved health outcomes among Indigenous Peoples (23, 24), supporting physical activity mentors as potential beneficial supports for Indigenous Peoples’ physical activity.

With a decrease in health status and PA participation, it is important to uncover factors which would improve participation amongst Indigenous Peoples (25). Currently within non-Indigenous populations, family support has shown a strong correlation to PA (22). The role of the family for Indigenous Peoples has been and continues to be impacted by colonization, and how family support impacts Indigenous Peoples’ PA participation is currently unknown. Recognizing the diversity of Indigenous identities, histories, and cultures within urban spaces in Canada, this study was conducted in partnership with community groups at the University of Saskatchewan, where multiple Indigenous identities are engaged, with sufficient numbers of specific identities to enable a distinctions-based approach. Therefore, the purpose of this study was to compare the physical activity of Indigenous adults in Saskatchewan with and without family/friend support of PA.

## Materials and methods

### Ethics & community engagement

To ensure the values of the participating Indigenous communities were prioritized during the research, an Indigenous Advisory Committee comprising of members from the Indigenous communities at the University of Saskatchewan (USask) was engaged and collaborated throughout the research process. The Indigenous Advisory Committee included an Indigenous undergraduate student, graduate student, faculty member, staff member, and an Elder, who were all selected by the partnering Indigenous groups, including the Indigenous Graduate Student Association, Indigenous Student Achievement Program, staff at the Aboriginal Students’ Centre and other USask Indigenous groups. An Elder is a leader and educator within their community who is respected and recognized as an important knowledge holder and maintains roles and knowledge of culture and ceremony of their community (26), This collaboration facilitated the research using a culturally sensitive way to examine how family support variables applicable to Indigenous Peoples influence PA.

The Usask Behavioural Research Ethics Board approved the research project. Before obtaining ethical approval from the USask Behavioural Research Ethics Board, the study design and measures were developed in partnership with the Indigenous Advisory Committee. The research team strove to balance being respectful to the needs and wishes of the Indigenous community, which was informed by the Indigenous Advisory Committee, while maintaining the standards of scientific procedures. The Indigenous Advisory Committee was integral in developing the methodology for the project. The interpretation of results from the study was discussed at a lunch and learn session with the Indigenous community and Advisors at the Aboriginal Students’ Centre at USask, the Gordon Oakes Red Bear Student Centre, where there were around 100 attendees. The Indigenous Advisory Committee were invited as authors on the publications of the study, but they all declined. Two other studies were conducted in parallel with some of the same participants, evaluating other aspects of Indigenous ways of life and PA, using the same methodology (17–19).

### Study design & participants

Participants were recruited through the assistance of partnering Indigenous groups and through PAWS, the USask web portal. Partnering Indigenous groups shared the invitation to participate through their member lists while they highlighted the relevance of the study to their community members. Emails regarding recruitment were sent out to all USask Indigenous identifying staff and faculty, and was shared through the Indigenous student centre email newsletter between 2018 and 2019. The advertisements for participation on PAWS, the university specific online bulletin board that appears when students and employees login to access university web services and platforms, and were also on various physical bulletin boards across USask. These advertisements engaged both targeted email lists of Indigenous staff and students, and university specific recruitment to the broader campus community, reaching Indigenous individuals who may not have self-selected to be included on Indigenous email lists. Eligible participants had to be at least 18 years old and self-identified as Indigenous. Informed consent was gathered from all participants before completing of any measures within this study. Participation occurred through an anonymous online survey (SurveyMonkey Inc., San Mateo, California, USA). Participants were provided the opportunity to be entered in a draw for one of six $50 gift cards to a local Indigenous store. The consent forms for this study were signed online at the start of the questionnaire.

Participants included students, staff, and faculty, including students from a range of years of study, degree types, and those who were living at home or living independently either on or off campus. This study was conducted at the University of Saskatchewan, located in Saskatoon, one of the sunniest cities in Canada (27). Weather in Saskatoon varies from long, comfortable summers with daytime temperatures ranging from 20˚C to 35˚C, to winters with abundant snowfall and daytime temperatures ranging from 5˚C to −30˚C (27).

### Measures

The Indigenous identities were determined based on participants’ responses to the questions regarding Indigenous groups and nations. Initially, there was a multiple-choice questionnaire regarding Indigenous groups, including: First Nations, Métis, Inuit, and other Indigenous Peoples. Then an open-ended question asked participants to identify and specify their Indigenous affiliations and/or nation. An open-ended question collected age and sex was reported as male, female or other/undefined. We collected self-identified gender, but due to small sample sizes of individuals reporting gender that did not align with sex, we chose not to report gender to maintain anonymity of participants. Marital status was reported as married/common-law, divorced, separated, widowed, or single/never married. Employment status options were student, temporarily unemployed, paid part-time or paid full-time. Level of education was reported as some high school, high school diploma, vocational school or some college, college/university degree or professional or graduate degree.

The definition of PA was provided including any play, work, transportation, household chores and recreation, and Indigenous-based examples such as lacrosse and dancing. The Godin Shepard Leisure Exercise Questionnaire (GSLEQ) was used to measure weekly PA (28), as previously used with Indigenous communities (29). Moderate (MPA) and vigorous PA (VPA) were calculated from reported minutes and frequencies of exercise, an overall GSLEQ score was calculated, and an overall weekly exercise frequency (WEF) was determined.

The Family Influence on Physical Activity questionnaire was used to record the extent and forms of family/friend support of PA (30, 31). This questionnaire asks: (1) How many days per week on average do your families/friends encourage you to engage in physical activity? (2) How many days per week on average do your families/friends watch you engaging in physical activity? (3) How many days per week on average do your families/friends involve themselves in your activities, that is, being physically active together with you? (4) My family/friends are physically active. Response options for these questions included (1) never or less weekly; (2) 1–2 days/week; (3) 3–4 days/week; (4) more than 5 days per week, and were collapsed to combine responses 1–2 as a “no” group, and responses 3–4 as a “yes” group. During initial conversations while developing the project, an additional question regarding role models was added on the recommendation of the Indigenous individuals at USask: (5) did you have a physical activity mentor/role model in your life? This included response options of yes or no.

### Statistical analysis

Analyses conducted were discussed with the Indigenous Advisory Committee to ensure respectful, useful, and ethical results. A distinctions-based approach was used, examining Métis and First Nations experiences separately. An analysis of Cree/Nehiyawak First Nations specifically was also included. Analyses of other First Nations identities were not conducted due to small sample sizes. An overall analysis of all responding Indigenous participants was also included to reflect the needs and supports of the USask community, where many distinct and diverse Indigenous Peoples are served. Statistical analysis comparing Indigenous identities was not performed on the recommendation of the Indigenous Advisory Committee, as they felt it did not add to the meaning or usefulness of the findings.

Mean differences were examined within each Indigenous identity with the significance set at *p* < 0.05. Data analysis was conducted using SPSS version 28.0. Statistical analysis included independent one-tailed *t-*tests. All assumptions for the independent *t-*tests were examined and met. Complex regression analyses were not conducted due to the sample size. This form of analysis was selected to reflect the distinctions-based approach, sample size, and community direction of the research question.

## Results

From the 140 participants who consented to participate in the study, 124 participants completed the survey (89%) from September 2018 to March 2019. As presented in Table 1, the majority of the participants were female and of Métis or First Nations identities. Of the First Nations participants, 67.5% identified as Cree/Nehiyawak. Participants ranged from 18 to 63 years of age.

**Table 1:** Characteristics of indigenous participants from University of Saskatchewan 2018–2019, by indigenous identity, *n* (%).

	Indigenous			
	Total	Métis	First Nations	
			All	Cree/Nehiyawak
N	123	40	80	54
Age, Years ± SD	30 ± 11	28 ± 12	31 ± 11	30 ± 12
Sex at birth				
Female	93 (75.2)	31 (75.6)	63 (77.8)	42 (77.8)
Male	30 (25.8)	9 (25.4)	17 (23.2)	12 (23.2)
Marital status				
Single, never married	51 (40.8)	14 (34.1)	35 (43.2)	24 (44.4)
In relationship	28 (22.4)	16 (39)	12 (14.8)	7 (13)
Married	18 (14.4)	4 (9.8)	14 (17.3)	9 (16.7)
Common-law	14 (11.2)	2 (4.9)	12 (14.8)	8 (14.8)
Separated/Divorced	11 (8.8)	4 (9.8)	6 (7.4)	5 (9.3)
Employment status				
Student	73 (58.4)	23 (56.1)	48 (59.3)	36 (66.7)
Paid part-time	12 (9.6)	4 (9.8)	8 (9.9)	3 (5.6)
Paid full-time	34 (27.2)	12 (29.3)	21 (25.9)	12 (22.2)
Education status				
High school	29 (23.2)	12 (29.2)	44 (54.3)	13 (24.1)
Some post-secondary	40 (32.1)	12 (29.3)	27 (33.3)	20 (37.0)
College/University degree	32 (25.6)	9 (22.0)	21 (25.9)	14 (25.9)
Professional/graduate degree	22 (17.6)	8 (19.5)	14 (17.3)	7 (13.0)

N, number of participants; SD, standard deviation; Percentages not add to 100% due to missing data or small sample sizes of some categories not listed to maintain confidentiality.

### Métis participants

**Figure 1:**
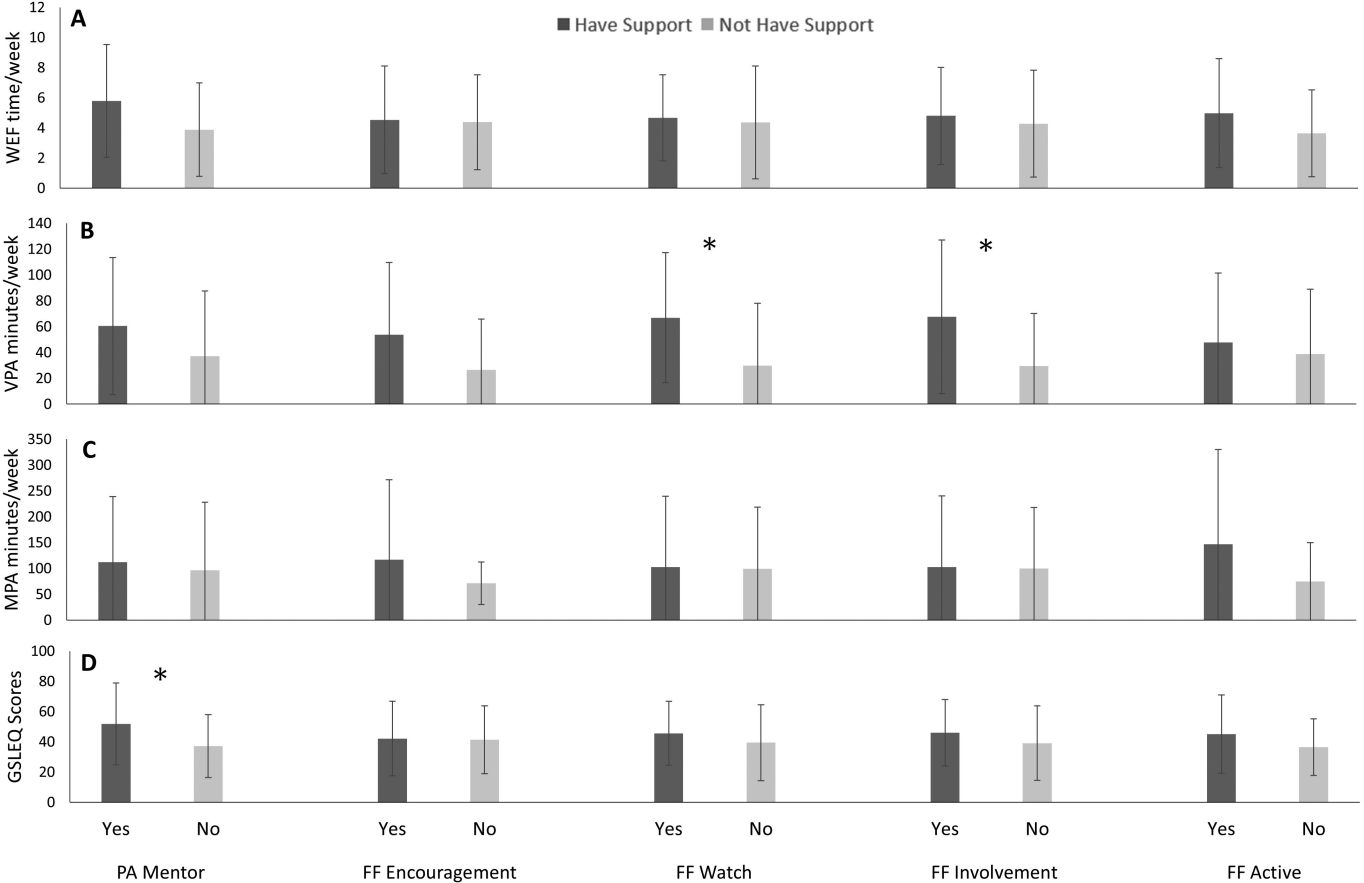
Weekly exercise freqeuncy (WEF; A), vigorous exercise (VPA; B), moderate exercise (MPA; C), and Godhin-Shephard leisure-time exercise Questionnaire scores (GSLEQ; D) scores from Métis participants from University of Saskatchewan, 2018–2019.

As outlined in Figure 1, Métis participants with family/friends involvement in PA reported greater VPA than those without family/friend involvement in PA (*p* = 0.01). Further, Métis participants with family/friends who watch them engage in PA report greater VPA in comparison to those who do not have family/friends watching them engage in PA (*p* = 0.01). Métis participants with PA mentors, a mentor or role model to support, advise, or demonstrate regular PA, reported greater GSLEQ scores than those who did not have PA mentors (*p* = 0.04).

### First Nations participants

**Figure 2:**
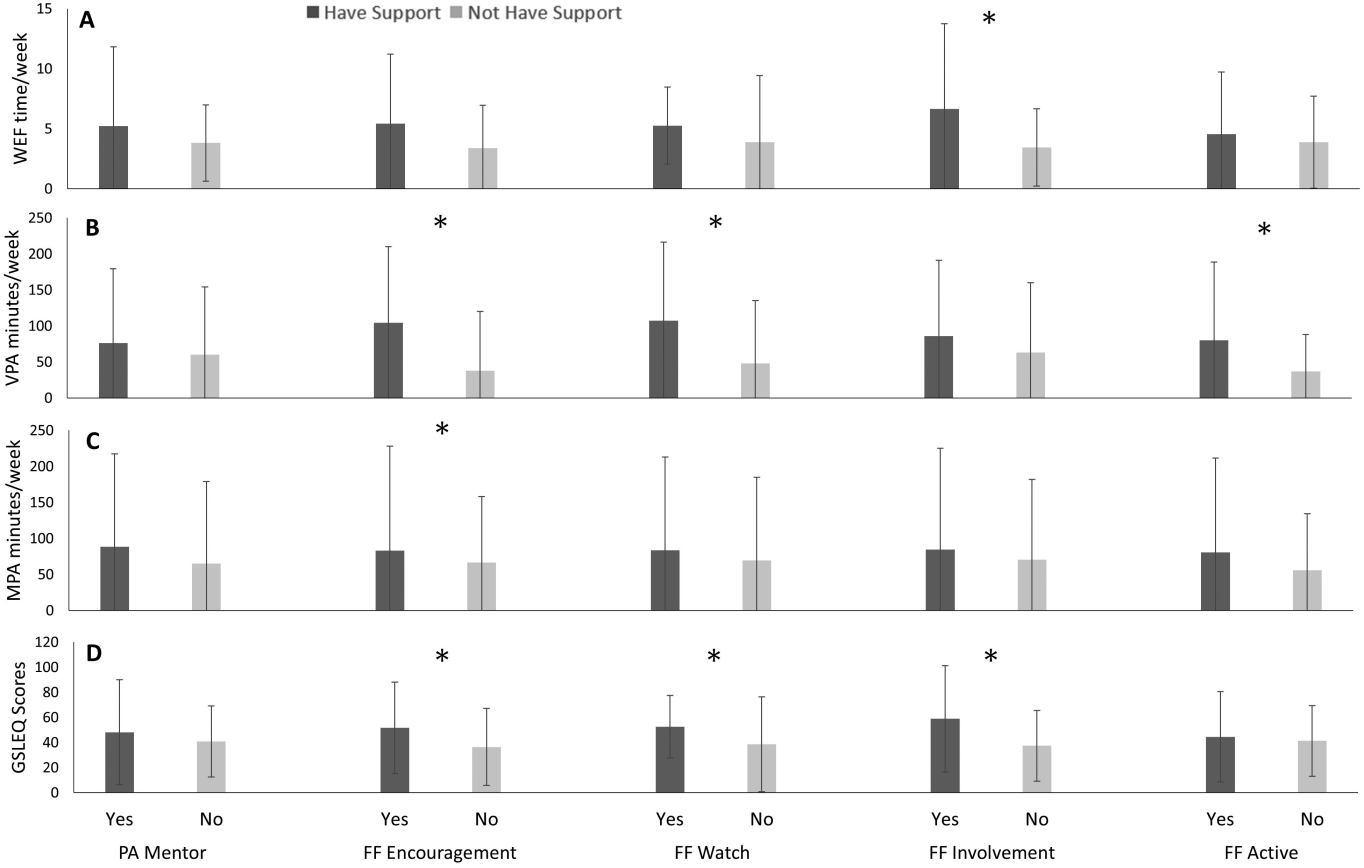
Weekly exercise freqeuncy (WEF; A), vigorous exercise (VPA; B), moderate exercise (MPA; C), and Godhin-Shephard leisure-time exercise Questionnaire scores (GSLEQ; D) scores from First Nations participants from University of Saskatchewan, 2018–2019.

Among First Nations participants, shown in Figure 2, those with family/friends’ encouragement of PA reported greater VPA (*p* = 0.002), WEF (*p* = 0.04) and GSLEQ scores (*p* = 0.03) than those without family/friends’ encouragement of PA. First Nations participants with family/friend involvement in PA reported greater WEF (*p* = 0.01) and GSLEQ scores (*p* = 0.01) in comparison to those without family/friends involved in their PA. Furthermore, First Nations participants with active family/friends reported significantly greater VPA (*p* = 0.01) in comparison to those without active family/friends. First Nations participants with family/friends who watch them engage in PA also have greater VPA (*p* = 0.01) and GSLEQ scores (*p =* 0.048) in comparison to participants who reported not having family/friends watch them engage in PA.

### Cree/Nehiyawak First Nations subgroup

**Figure 3:**
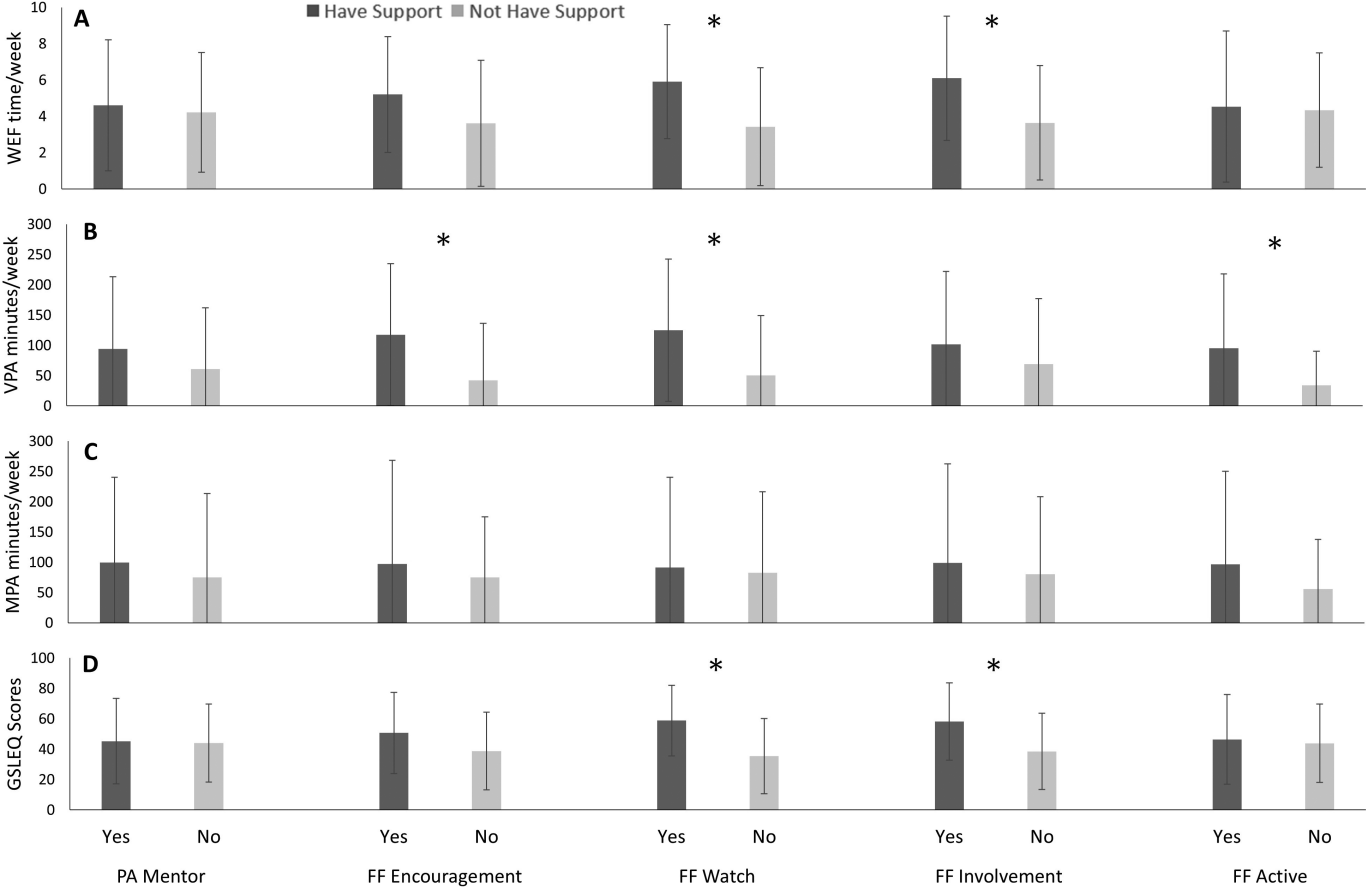
Weekly exercise freqeuncy (WEF; A), vigorous exercise (VPA; B), moderate exercise (MPA; C), and Godhin-Shephard leisure-time exercise Questionnaire scores (GSLEQ; D) scores from Cree/Nehiyawak participants from University of Saskatchewan, 2018–2019.

In Figure 3, Cree/Nehiyawak First Nations participants with family/friend encouragement of PA reported greater VPA, than those without family/friends’ encouragement of PA (*p* = 0.01).Cree/Nehiyawak participants with family/friend involvement in PA reported greater WEF (*p* = 0.01) and GSLEQ scores (*p* = 0.01) than participants who did not have family/friends involved in their PA.

### Indigenous participants

**Figure 4:**
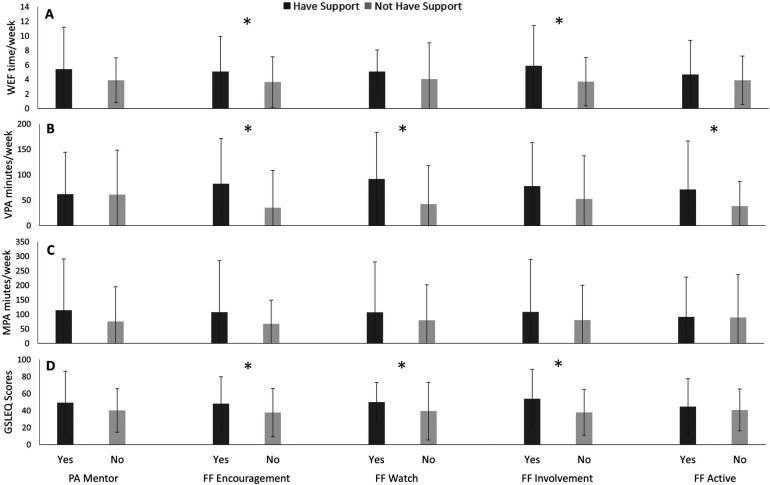
Weekly exercise freqeuncy (WEF; A), vigorous exercise (VPA; B), moderate exercise (MPA; C), and Godhin-Shephard leisure-time exercise Questionnaire scores (GSLEQ; D) scores from collective indigenous participants from University of Saskatchewan, 2018–2019.

From the collective Indigenous participants within this study, VPA was greater amongst those with family/friend encouragement of PA in comparison to the participants without family/friend encouragement of PA (*p* = 0.001), as seen in Figure 4. Similarly, Indigenous participants with family and friends who watch them engage in PA reported greater VPA (*p* = 0.001) and GSLEQ scores (*p* = 0.04) than those without family and friends who watch them engage in PA. Further, Indigenous participants with family/friends’ involvement in PA reported greater weekly exercise frequency (WEF), in comparison to those without this involvement in PA (*p* = 0.01). Greater GSLEQ scores were also reported from Indigenous participants with active family and friends compared to those without active family and friends (*p* = 0.01).

## Discussion

This research was significant in identifying greater PA among Indigenous Peoples with family/friend support of PA. These findings build on and are consistent with previous research identifying greater PA among Indigenous Peoples with greater perceptions of social support (18). Another important finding is the distinctiveness of results among different Indigenous identities, with Métis adults reporting greater PA when they have a PA mentor, while this finding was not identified among First Nations adults. Partnering with Indigenous communities at a university enabled an analysis of diverse a Indigenous population, representative of many urban centres, while also including sufficient numbers of participants from specific identities to enable a distinctions-based approach to this analysis.

Within Indigenous communities, a sense of belonging and support from on's community is a significant determinant of health. For Indigenous Peoples, social support is linked to health and wellness, and reinforces cultural identity (32, 33). Previous research has reported that thriving health is more common among Indigenous Peoples with greater social support (32). Among the general population, support from family and friends has been identified as a correlate of PA for adults, and support for physical activity from family/friends has been identified as a correlate of PA among children/youth (34). Furthermore, among adults in the general population, family/friends support for PA is associated with weight reduction (35, 36). Within Indigenous communities, family participation in PA particularly for youth, models positive behaviour, which is imprinted on children through adulthood (37). Within our study specifically, the greater PA among Indigenous Peoples with family/friends involvement in PA among multiple Indigenous identities, supports the strength of this finding.

Family/friend support in the form of PA mentors was associated with greater PA among Métis People specifically. The lack of PA difference among First Nations who have or do not have a PA mentor is consistent with previous findings among children and youth in the general population (34). However, among First Nations youth, having relatives help youth understand their culture is associated with greater PA among First Nations youth (38). Mentorship is an important component of many Indigenous cultures and integral to knowledge transfer and teachings (39). Indigenous mentorship is valuable for connecting people through culture and providing resources for mentees’ growth (39). Among First Nations youth, mentorship is associated with decreased weight gain and improved healthy living (40). These findings also highlight the distinctiveness and differences between Indigenous identities and the need for distinctions-based approaches to supporting Indigenous health and wellbeing. This connection to culture and mentorship, consistent with previous reports of greater cultural connectedness associated with greater physical activity support resourcing for Indigenous cultural connections as a potential means of improving physical activity for Indigenous Peoples (17). Future studies should evaluate interventions focusing on expanding cultural connectedness, knowledge, and engagement to evaluate PA changes.

The high levels of VPA reported in this study contrast previous research among First Nations communities identifying participation predominantly in low-intensity PA (41). This may reflect the university campus setting of participants, reflecting a primarily young middle-aged adult population who have access to PA facilities and programs on post-secondary campuses and greater education associated with PA among Indigenous Peoples (42). University populations have been found to have to be highly active, with more than 70% of university students classified as highly active (43). However, physical activity behaviours among university populations are highly variable depending on the country and culture (44), highlighting that Indigenous Peoples experiences may be different from that of other university populations in Canada. Further understanding is required to better articulate the differences in PA among Indigenous Peoples who have post-secondary education and the influence of access to PA facilities and programs may have on PA behaviour.

This study explored Indigenous Peoples’ PA behaviour and how it is related to family/friend support, a previously unexplored concept. A strength of this research is the distinctions-based approach, identifying similar and unique modes of family/friends support of PA among distinct Indigenous identities. Partnership with the Indigenous community and oversight of an Indigenous Advisory Committee is an important strength in this work. Another strength of this study was the diverse perspectives and experiences of Indigenous Peoples represented. As participants representing and experiencing multiple Indigenous identities, and from different income, education, employment backgrounds are included, this sample is closer in representation to urban Indigenous populations. Evaluating PA based on self-reported measures has potential limitations. The university campus setting of this research may limit generalizability to Indigenous populations in other locations or settings. In 2021 just under half (49.2%) of Indigenous Peoples aged 25–64 years in Canada had completed a postsecondary qualification, which is below the rate for the non-Indigenous population in Canada (68.0%) (45). Further, we did not ask which academic unit participants registered with; participants from more health-focused disciplines may have been more interested in participating. Additionally, if students also worked, they may not have been included in either student, or employed categories of our demographic assessment, limiting the consistency of student and employment characteristics. Limitations of this study also include a sex bias in the sample, with more females completing the survey, though this is consistent with a gender bias of more Indigenous women than men in higher education (46).

Family/friends support for PA, including involvement in PA and encouragement of PA are associated with greater PA for collective Indigenous populations, and specifically Métis, First Nations, and Cree/Nehiyawak First Nations. Specifically, among Métis, participants with PA mentors also reported greater PA than those without PA mentors. Family/friends support for PA is an enabler of PA for Indigenous Peoples, though specific identities experience differences in the components of family/friend support for PA. Future studies should evaluate how family, friend, and community support of PA influences PA of Indigenous Peoples living in rural, remote, and on-reserve locations, and those who are not engaged in post-secondary education and careers. Additionally, evaluating sex- or gender-specific experiences among Indigenous Peoples would also be important.

## Data Availability

The datasets presented in this article are not readily available because the participants of this study, and the community partners, did not give written consent for their data to be shared publicly, so due to the sensitive nature of the research partnering with an Indigenous community, supporting data is not available. Requests to access the datasets should be directed to heather.foulds@usask.ca.
